# Editorial: Quality assurance and workflow optimization for the diagnosis and treatment of head and neck cancer

**DOI:** 10.3389/fonc.2024.1497257

**Published:** 2024-10-08

**Authors:** Olgun Elicin, Sefik Hosal

**Affiliations:** ^1^ Department of Radiation Oncology, Inselspital, Bern University Hospital and University of Bern, Bern, Switzerland; ^2^ Department of Otolaryngology-Head & Neck Surgery, Atılım University Faculty of Medicine, Ankara, Türkiye

**Keywords:** head and neck squamous cell carcinoma, workflow, artificial intelligence, quality assurance, information system

The management of head and neck cancer (HNC) requires a coordinated multidisciplinary approach, integrating advanced imaging, pathology, medical and radiation oncology, surgery, and supportive care. This Research Topic of *Frontiers in Oncology* emphasizes the critical need for quality assurance and workflow optimization in diagnosing and treating HNC. It addresses the challenges posed by increasingly complex healthcare systems and the necessity for effective electronic health record (EHR) management. The following articles contribute valuable insights to our understanding of these issues.

**1. Safety and Usability of Oncology Information Systems:** Cihoric et al. conduct a systematic review assessing the safety and usability of oncology information systems (OIS) used in clinical practice. They identify significant challenges related to documentation time, communication barriers, and data integration issues impacting clinical workflows. Despite the recognized importance of EHR usability and safety, comprehensive data is lacking, highlighting the need for further research and development. The authors argue for the involvement of end-users in the design and continuous evaluation of these systems to enhance their usability and flexibility, which are critical for effective clinical decision-making and patient safety.

**2. Role of Artificial Intelligence (AI) in Patient Education:** Kuşcu et al. explore the potential of ChatGPT as a tool for patient education and clinical decision support in the context of HNC. By evaluating the accuracy and reliability of ChatGPT’s responses to 154 HNC-related questions sourced from professional societies, patient support groups, and social media, they found that the AI model provided “comprehensive/correct” answers to 86.4% of queries and exhibited a high reproducibility rate of 94.1%. While the model shows substantial promise, it should not replace professional medical advice due to its limitations and reliance on data up to 2021. The authors suggest that with further improvements, AI tools like ChatGPT could significantly support clinicians and enhance patient understanding, especially in resource-limited settings.

**3. Prediction of Nodal Status:** Chen et al. focus on developing and validating a nomogram to predict cervical lymph node metastasis in patients with HNC. Recognizing lymph node metastasis as a critical prognostic factor, they utilized clinical, imaging, and pathological data from 272 patients to construct a predictive model in a low-cost setting. The nomogram demonstrated high predictive accuracy with a C-index of 0.91 and area under the curve values of 0.953 and 0.938 for the training and validation cohorts. This model aids in patient risk stratification and treatment planning, highlighting the potential for integrating imaging and pathological features into predictive models to enhance precision in cancer treatment strategies.

**4. Improving and Structuring Interdisciplinary Communication in Pre-Treatment Setting:** Crosetti et al. propose a structured protocol to enhance the planning and execution of open partial horizontal laryngectomy for laryngeal cancer. The manuscript presents a checklist of 20 critical questions that surgeons should discuss with radiologists to ensure thorough preoperative evaluation and optimize surgical outcomes. By covering key anatomical structures and tumor extents, the protocol facilitates informed decisions regarding surgical approaches. This approach not only improves the precision of preoperative planning but also serves as an educational tool for less experienced clinicians, standardizing evaluation processes and improving oncological and functional outcomes.

**5. Decision-Making in High-Volume Multidisciplinary Team Meetings (MDTs):** Hendrickx et al. examine the impact of MDTs on treatment decisions in HNC care within a high-volume oncological center. The retrospective study emphasizes the importance of thorough re-evaluation through physical examinations, diagnostic imaging, and pathology reviews, often leading to adjustments in TNM classifications and treatment plans. Key findings reveal that management recommendations changed in 22% of cases after MDT discussions at the HNOC, with major changes in 16% of cases, primarily involving treatment intensification or de-intensification. The authors advocate for mandatory MDT discussions for all patients treated at lower-volume centers to optimize outcomes in HNC management.

**6. Workflow Optimization for Pathology Workup of Transoral Laser Microsurgery:** Meulemans et al. introduce a new standardized pathology workup protocol for glottic cancer treated with transoral laser microsurgery (TLM). This prospective study included 96 patients and aimed to improve the evaluability of surgical margins, which are crucial for determining the need for additional treatments and predicting patient outcomes. The protocol, involving the oriented fixation of TLM specimens on pig liver slices, enhances the accuracy of deep margin evaluation, significantly reducing false-positive assessments. While it did not significantly change overall survival or disease-free survival, it allows for better-informed decisions regarding adjuvant therapy and follow-up intensity, potentially optimizing patient management and avoiding complications through unnecessary treatments.

This Research Topic provides a comprehensive overview of the current state of HNC management, emphasizing the need for improved quality assurance and workflow optimization. The integration of advanced technologies, such as AI and precision medicine, is crucial for enhancing diagnostic accuracy and treatment efficacy. Moreover, the role of EHR systems in streamlining clinical workflows is vital to ensure seamless communication and coordination among multidisciplinary teams. It is imperative to continue fostering collaboration across specialties and investing in technological innovations that support personalized medicine. By addressing the challenges of system interoperability and user engagement, healthcare providers can enhance the quality of care and improve outcomes for patients with HNC. Transparency and collaboration between the industry and healthcare providers play a critical role in achieving these goals.

In conclusion, this Research Topic underscores the need for continuous improvement and adaptation in cancer care practices, highlighting the role of technology and multidisciplinary collaboration in driving advancements in the field. We hope these contributions will inspire further research and innovation to optimize the management of HNC and ultimately improve treatment outcomes, safety, and the satisfaction of patients and healthcare professionals.

